# Missed Kawasaki Disease With Coronary Aneurysms and Familial Hyperlipidemia: A Case Report

**DOI:** 10.1002/ccr3.71826

**Published:** 2026-01-12

**Authors:** Roddy Gergeus, Jewel Khannoura, Dalaa Sheikh Ali, Yara Sayed‐Ahmad, Fadi Khoury

**Affiliations:** ^1^ Al‐Baath University Homs Syria; ^2^ Tishreen University Latakia Syria; ^3^ University of Kalamoon Deir Atiyah Syria

**Keywords:** coronary aneurysm, coronary angiography, familial hyperlipidemia, HDL, Kawasaki disease, LDL

## Abstract

Atypical Kawasaki disease may be missed, leading to severe coronary complications. Physians should suspect Kawasaki disease in infants with prolonged fever and elevated inflammatory markers, even without the classic features, providing long‐term cardiac and lipid monitoring.

**Abbreviations**
AHA: American Heart Association
BMI: Body Mass Index
CAA: coronary artery aneurysm
CRP: C‐reactive protein
ECG: electrocardiogram
ESR: erythrocyte sedimentation rate
Hb: hemoglobin
HDL: high‐density lipoprotein
IVIG: intravenous immunoglobulin
KD: Kawasaki Disease
LAD: left anterior descending (Artery)
LDL: low‐density lipoprotein
LMCA: left main coronary artery
N%: neutrophil percentage
RCA: right coronary artery
TG: triglycerides
WBC: white blood cell

## 1 Introduction

Kawasaki disease (KD) is an acute vasculitis of childhood with unknown etiology. It most commonly affects children under 5 years of age and is more common in males and children of Asian descent [1].

The disease causes inflammation in medium‐sized arteries, especially the coronary arteries, and typically presents with prolonged fever and mucocutaneous changes, and lymph node involvement [1].

Without timely treatment, approximately 25% of patients may develop coronary artery aneurysms, which can lead to myocardial infarction, ischemic heart disease, or sudden cardiac death [2].

Classic KD is diagnosed based on clinical criteria including persistent fever for at least 5 days along with at least four of five characteristic features (mucocutaneous changes, conjunctival injection, polymorphous rash, extremity changes, and cervical lymphadenopathy) [2]. However, incomplete or atypical forms of KD may lack these hallmark signs, making early diagnosis challenging and increasing the risk of delayed treatment long‐term cardiovascular complications [3]. We report a rare case of missed diagnosis of Kawasaki disease in infancy, later complicated by giant coronary aneurysms and coexisting familial hyperlipidemia [4]. To our knowledge, this is one of the few documented cases highlighting the intersection of missed atypical KD and inherited dyslipidemia in a low‐resource setting.

## 2 Case History/Examination

A 12‐year‐old male presented to the emergency department with intermittent chest pain. He was later admitted to the cardiology department due to episodes of unstable angina attacks accompanied by diaphoresis. These symptoms predominantly occurred at night, frequently waking him from sleep, and were severe enough to restrict his daytime activities, including playing with peers. There was no known chronic medical condition, and his birth and developmental history were unremarkable. Physical examination revealed normal vital signs: temperature 36.7°C, heart rate 90 bpm, respiratory rate 17 breaths/min, and normal blood pressure. His height and weight were within age‐appropriate ranges, with a BMI of 22.5 kg/m^2^. Notably, he had acanthosis nigricans on the posterior neck, suggestive of insulin resistance.

His family reported a hospitalization at 7 months of age for a persistent fever lasting over 15 days.

## 3 Differential Diagnosis, Investigations and Treatment

During that time, no definitive diagnosis was made, and he was treated as a case of gastroenteritis. Laboratory tests during that episode showed progressive leukocytosis, fluctuating CRP, anemia, and pyuria (Table 1). There was no documented conjunctivitis, mucosal changes, or rash, and he did not meet the classic diagnostic criteria for KD at that time.

**TABLE 1:** Laboratory findings over time.

Parameter	At 7 months (hospitalization)	At 12 years (admission)	7‐month FU	10‐month FU	Reference range
WBC (/mm^3^)	20,000 → 47,000 → 27,300	12,300	—	—	4000–10,000
Neutrophils (%)	—	67%	—	—	40–70
Hemoglobin (g/dL)	10.55 → 8.7	13.5	—	—	12–16
CRP (mg/L)	128 → 95 → 183	2.18	—	—	< 10
ESR (mm/h)	—	36	—	—	< 20
Total Cholesterol (mg/dL)	—	279	101	279	< 200
LDL (mg/dL)	—	181	55	181	< 100
HDL (mg/dL)	—	39	30	39	> 40
TG (mg/dL)	—	164	88	164	< 150
Urinalysis	Pyuria (40–50 WBCs/HPF)	—	—	—	—

Further testing revealed that the child, along with his siblings, had familial hyperlipidemia, confirmed by lipid profile results (Table 1). ECG was normal. However, echocardiography revealed three coronary aneurysms involving the left and right coronary arteries and the left anterior descending artery (Table 2).

**TABLE 2:** Echocardiography and coronary angiography findings.

Coronary site	Initial echo (12 years)	Coronary angiography	7‐month FU	10‐month FU	AHA classification^a^
LCA (LMCA)	8.8 mm	Aneurysmal dilation in proximal LMCA	5.0 mm	—	Giant (> 8 mm)
LAD	5.0 mm	Mid‐to‐distal LAD dilation	—	—	Medium (5–8 mm)
RCA	4.5–5.0 mm	Proximal to segment 2 dilation	—	6.1 mm	Medium/Large

^a^ AHA: small < 5 mm, medium 5–8 mm, giant > 8 mm.

**FIGURE 1:**
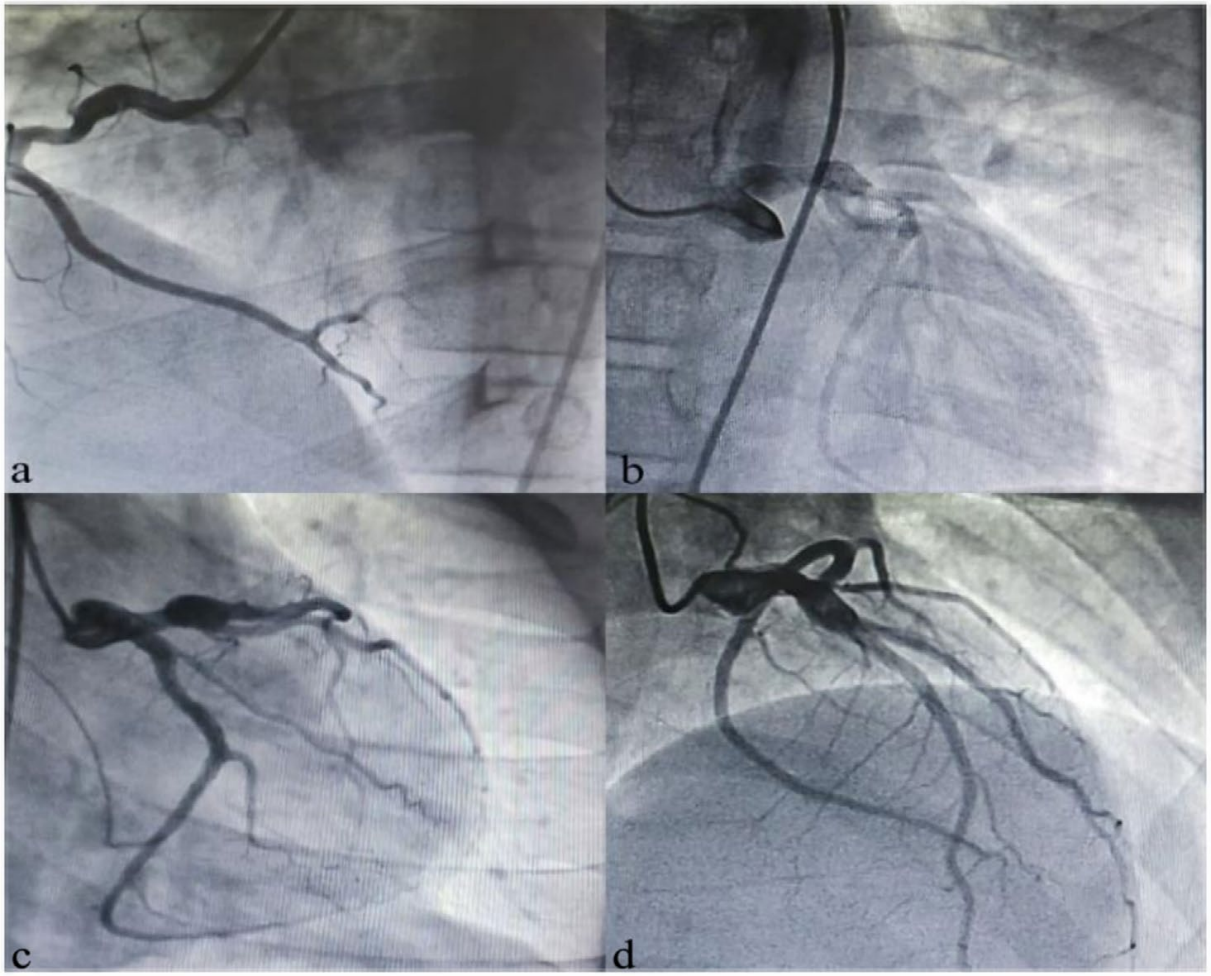
Coronary angiography showing aneurysmal dilations. Coronary angiography at age 12 years demonstrating aneurysmal dilations in the proximal left main coronary artery (LMCA), mid‐to‐distal left anterior descending artery (LAD), and right coronary artery (RCA).

Coronary angiography was performed, confirming aneurysmal dilations in the proximal left main coronary artery, the mid‐to‐distal left anterior descending artery, and the right coronary artery (Table 2, Figure 1). The initial echocardiogram revealed a left coronary artery aneurysm measuring 8.8 mm, with additional dilations in the LAD (5 mm) and RCA (4.5–5 mm).

Based on the retrospective clinical and imaging findings, this child was ultimately diagnosed with Kawasaki disease (KD), with coronary aneurysms as complications of the misdiagnosed illness during infancy. A diagnosis of familial hyperlipidemia was also established due to the elevated lipid profile and similar findings in two siblings. Upon admission, the patient was started on aspirin, clopidogrel, and rosuvastatin (Table 3).

**TABLE 3:** Treatment course and adjustments.

Medication	Initial dose	Adjustments	Purpose	Outcome
Aspirin	81 mg/day	No change	Antiplatelet	Maintained aneurysm stability
Clopidogrel	37.5 mg/day	↑ to 75 mg/day (weight‐adjusted)	Antiplatelet	Prevented thrombosis, maintained CAA regression
Rosuvastatin	20 mg/day	↓ to 10 mg (after lipid improvement) → ↑ back to 20 mg (after lipid relapse)	Lipid‐lowering	Improved cholesterol initially, relapse at 10 months

## 4 Conclusion and Results (Outcome and Follow‐Up)

**FIGURE 2:**
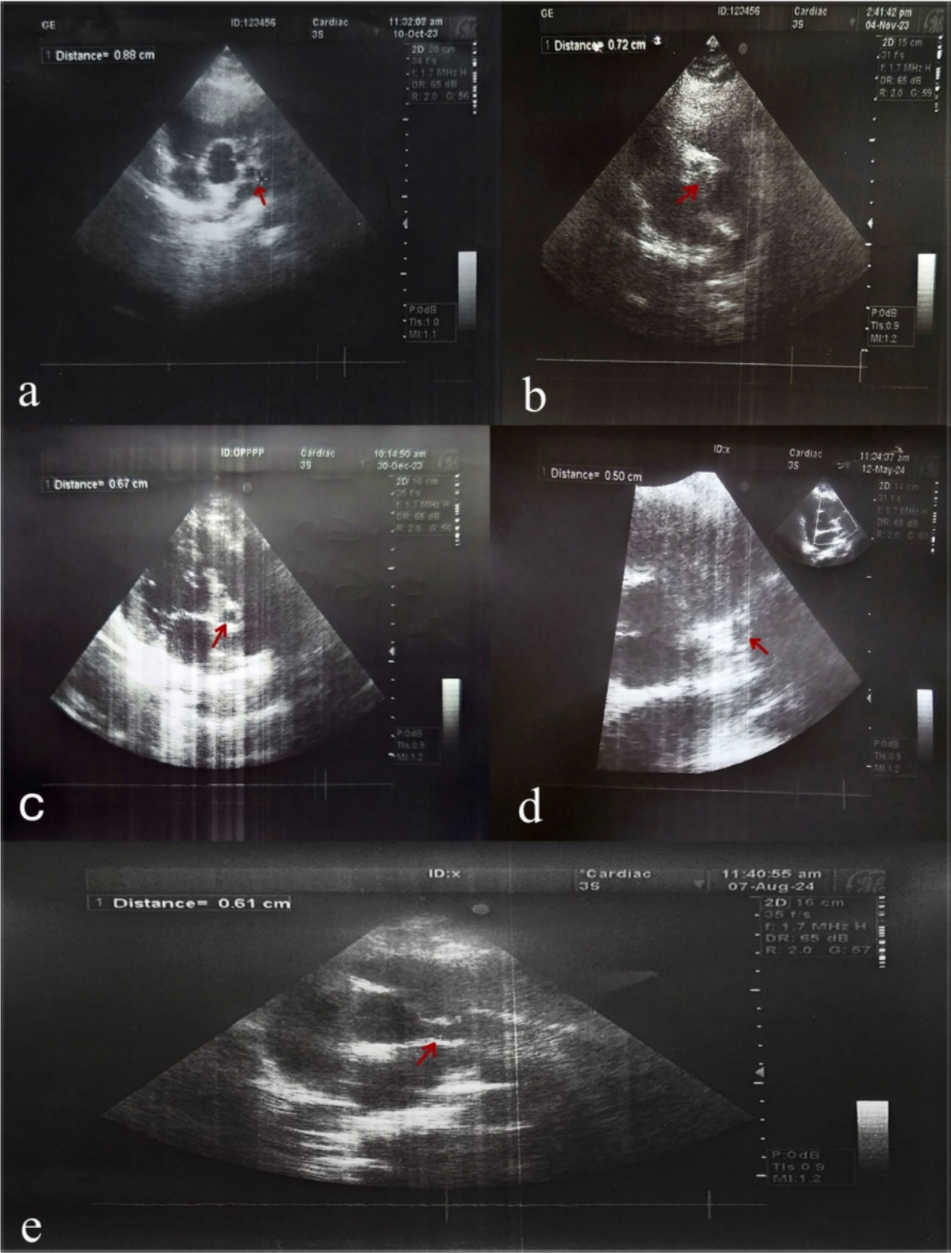
Serial echocardiography follow‐up. Echocardiographic measurements showing changes in the left coronary artery aneurysm over time, with regression from 8.8 mm to 5.0 mm at 7 months, followed by an increase to 6.1 mm at 10 months.

At 7 months of treatment, follow‐up echocardiography revealed a decrease in the size of the left coronary artery aneurysm from 8.8 mm to 5 mm (Figure 2), and his lipid profile showed significant improvement (Tables 1 and 2). The rosuvastatin dose was reduced, and clopidogrel was adjusted for weight (Table 3). At the 10‐month follow‐up, echocardiography showed a slight increase in the RCA aneurysm size, accompanied by worsening lipid profile results (Tables 1 and 2). Rosuvastatin was increased back to 20 mg. The patient is currently stable, with resumption of daily activities and ongoing cardiac and lipid monitoring.

This case highlights that Kawasaki disease (KD) may present atypically, leading to missed diagnoses and delayed treatment with serious cardiovascular sequelae. Our patient developed giant coronary aneurysms due to untreated KD in infancy, with the additional risk factor of familial hyperlipidemia. The case emphasizes the importance of considering atypical KD in infants with prolonged fever, ensuring timely diagnosis and intervention, and improving access to standard therapies such as intravenous immunoglobulin (IVIG), particularly in low‐resource settings.

## 5 Discussion

Kawasaki disease (KD) is an acute, self‐limited vasculitis that predominantly affects children under 5 years of age, with about 80% of cases occurring in this age group [5]. Recent studies have demonstrated significant geographic and seasonal variation in KD incidence, with the highest rates reported in East Asia. Peaks such as winter and early spring, support the hypothesis of an infectious or environmental trigger [6, 7]. The exact cause of KD remains unknown. Current evidence suggests that genetic susceptibility and autoimmune mechanisms play important roles, while infectious agents and environmental factors may act as external triggers [7, 8]. One leading hypothesis is that KD may be driven by a single, unidentified respiratory virus rather than multiple pathogens or toxins. Supporting this idea, intracytoplasmic inclusion bodies—typical of viral replication—have been consistently observed in KD‐affected tissues across decades and geographic regions [9].

KD is diagnosed based on clinical criteria; however, incomplete or atypical forms in infants may lack sufficient manifestations, increasing the risk of misdiagnosis and cardiac damage [10]. Our patient was hospitalized at 7 months with persistent fever and elevated inflammatory markers but was treated as gastroenteritis, likely because classical signs, such as conjunctivitis or rash were absent. This diagnostic delay allowed the development of significant coronary complications, which became evident years later [10].

Giant coronary aneurysms are rare but serious complications of KD. The American Heart Association classifies CAAs as small (< 5 mm), medium (5–8 mm), and giant (> 8 mm) [2]. Our patient developed a giant aneurysm (8.8 mm) in the left coronary artery, along with additional aneurysms in the LAD and RCA, illustrating the consequences of untreated KD in infancy. He was also found to have familial hyperlipidemia, which increased his cardiovascular risk. Dyslipidemia is a known risk factor to long‐term complications, and KD as well as abnormal lipid profiles are each linked to increased cardiovascular risk [11]. Children with a history of KD frequently demonstrate lipid abnormalities on follow‐up, including reduced HDL‐C and unfavorable LDL‐P/HDL‐P ratios [12]. In our case, dyslipidemia remained despite statin therapy, further increasing the risk of premature atherosclerosis.

Long‐term management targeted both coronary complications and dyslipidemia. Due to limitations in healthcare resources, IVIG the first‐line treatment for KD was not available at the time of diagnosis. Instead, the patient was managed with aspirin, clopidogrel, and rosuvastatin (Table 3). Despite the absence of IVIG, the coronary aneurysms regressed over time, particularly in the left coronary artery, which decreased from 8.8 mm to 5 mm. This regression suggests potential benefit from long‐term antiplatelet and lipid‐lowering therapy, though IVIG remains the standard treatment for acute KD [2]. From this experience, several key learning points emerge.

Atypical KD should be considered in infants with prolonged fever, even without the classical signs [13]. Delayed diagnosis can lead to irreversible cardiac sequelae, including giant CAAs. The coexistence of familial hyperlipidemia further increases long‐term cardiovascular risk [13]. While IVIG is the gold standard, alternative strategies may provide some benefit in resource‐limited settings [14].

### 5.1 Case Presentation

The patient presented with persistent fever, leukocytosis, anemia, and elevated inflammatory markers. Laboratory results and follow‐up lipid profiles are summarized in Table 1. Echocardiography and coronary angiography revealed multiple coronary aneurysms, detailed in Table 2.

## 6 Discussion

The patient's management involved multiple medications, with adjustments made over time based on lipid profile and coronary outcomes. The treatment course is detailed in Table 3.

## Author Contributions

**Roddy Gergeus:** data curation, formal analysis, investigation, project administration, validation, visualization, writing – original draft. **Jewel Khannoura:** data curation, formal analysis, investigation, resources, visualization, writing – original draft. **Dalaa Sheikh Ali:** data curation, formal analysis, investigation, methodology, resources, visualization, writing – original draft. **Yara Sayed‐Ahmad:** conceptualization, data curation, investigation, project administration, visualization, writing – original draft, writing – review and editing. **Fadi Khoury:** formal analysis, investigation, supervision, validation, visualization.

## Funding

The authors have nothing to report.

## Consent

Written informed consent was obtained from the patient's legal guardian for the publication of this case report and any accompanying images.

## Data Availability

The data that support the findings of this study are available from the corresponding author upon reasonable request.
